# Epidemiology and long-term disease burden of herpes zoster and postherpetic neuralgia in Taiwan: a population-based, propensity score-matched cohort study

**DOI:** 10.1186/s12889-018-5247-6

**Published:** 2018-03-20

**Authors:** Wan-Hsuan Lu, Chih-Wan Lin, Chen-Yu Wang, Liang-Kung Chen, Fei-Yuan Hsiao

**Affiliations:** 1 Graduate Institute of Clinical Pharmacy, College of Medicine, National Taiwan University, Room 220, 33, Linsen S. Rd, Taipei, 10050 Taiwan; 2 School of Pharmacy, College of Medicine, National Taiwan University, Taipei, Taiwan; 3 Aging and Health Research Center, National Yang Ming University, Taipei, Taiwan; 4 Center for Geriatrics and Gerontology, Taipei Veterans General Hospital, Taipei, Taiwan; 5 Department of Pharmacy, National Taiwan University Hospital, Taipei, Taiwan

**Keywords:** Herpes zoster, Postherpetic neuralgia, Epidemiology, Disease burden

## Abstract

**Background:**

The objectives of this study were to characterize the burden of herpes zoster, as well as the longitudinal and incremental changes of healthcare service utilization among individuals with herpes zoster and postherpetic neuralgia (PHN) compared to those without.

**Methods:**

Using the National Health Insurance Research Database (NHIRD), we established a herpes zoster cohort of people diagnosed with herpes zoster between 2004 and 2008 as study cases. Another subset of the NHIRD, which was randomly selected from all elderly beneficiaries between 2004 and 2008 served as a non-herpes-zoster elderly control pool. Each case was then assigned one matched control according to age, gender, index date and propensity score. PHN cases were defined as those with persisting pain for more than 90 days after the onset of herpes zoster.

**Results:**

Between 2004 and 2008, about 0.6 million patients were newly diagnosed with herpes zoster. The incidence increased with age, and most cases were identified during the summer period. Herpes zoster cases were found to have higher consumption of all types of healthcare services in the first year after the index date. Such increases were particularly obvious for patients with PHN, who showed incremental increases on average of 16.3 outpatient visits, 0.4 emergency room visits and 0.24 inpatient admissions per year.

**Conclusions:**

The incidence of herpes zoster increased with age and changed according to the seasons. Patients with herpes zoster were associated with higher healthcare utilization and this increase in healthcare utilization was most obvious for herpes zoster patients with PHN.

**Electronic supplementary material:**

The online version of this article (10.1186/s12889-018-5247-6) contains supplementary material, which is available to authorized users.

## Background

Herpes zoster is a reactivated condition of varicella zoster virus (VZV) infections, which has resulted in considerable morbidity and mortality worldwide, especially in the elderly population [1, 2]. Postherpetic neuralgia (PHN) is the most common and important complication related to herpes zoster infections, and is associated with long-term suffering and poor quality of life in affected populations [3]. A number of therapies have been used to manage herpes zoster and PHN. However, those therapeutics usually show limited efficacy, and none of them can prevent the recurrence of this disease except vaccinations [4].The herpes zoster vaccine has demonstrated efficacy in reducing the incidence and disease burden of herpes zoster and PHN in randomized, controlled clinical trials among older adults and patients age 50–59 years old [5, 6]. Similar results have been shown in large-scale post-marketing studies [7].

Previous studies have explored the epidemiology and associated healthcare service utilization of herpes zoster [8–22]. However, several essential limitations still need to be overcome. Many of the currently existing studies have focused on the estimation of short-term healthcare service utilization (within 6 to 12 months after the diagnosis of herpes zoster) [15–19] or only some selected high-risk populations [20, 21]. Moreover, currently, only healthcare service utilization “directly” related to herpes zoster (such as admissions with a primary diagnosis of herpes zoster) have been included in the estimation of disease burden [9, 12], while the overall burden including complication-oriented healthcare utilization among those frail patients may be underestimated. Furthermore, the majority of previous studies have lacked properly designed control groups, which may limit appropriate estimations about the “incremental” burden of herpes zoster [22].

Therefore, population-based studies, addressing specific methodological concerns, are needed to quantify the disease burden of people with herpes zoster and are essential for strategizing future prevention and vaccination. The objectives of this study were to characterize the burden of herpes zoster, as well as the longitudinal and incremental changes of healthcare service utilization among individuals with herpes zoster and PHN compared to those without.

## Methods

### Data source

Launched in 1995, Taiwan’s National Health Insurance (NHI) system is a mandatory, single-payer health insurance program organized by the government and operated by the National Health Insurance Administration (NHIA) in Taiwan. It provides comprehensive benefits including inpatient care, ambulatory care, dental care and prescription drug coverage to their beneficiaries. With approximately 23 million insured, it covers over 99% of the population of Taiwan.

The NHIA and National Health Research Institutes (NHRI) in Taiwan maintain a database, the National Health Insurance Research Database (NHIRD), which consists of claims and transactions for all covered services utilized by patients enrolled in the NHI program. This database includes information on demographic, clinical, medical resource utilization (outpatient and inpatient visits), costs of services, and treatment patterns. All traceable personal identifiers are removed from the database to protect patient confidentiality. The completeness and accuracy of the NHI claims databases are also ensured by the NHIA and NHRI of Taiwan [23].

### Ethical statement

Since the identification numbers of all subjects in the National Health Insurance Research Database (NHIRD) were encrypted to protect the privacy of the individuals, this study was exempted from full review by the Institution Review Board of the National Taiwan University Hospital and informed consents were waived (201411050W).

### Study population

In this study, we used a specific subset of the NHIRD, which consisted of all claims of patients with a diagnosis of herpes zoster between 2004 and 2008, as the herpes zoster cohort. By using this cohort, we investigated the epidemiology of herpes zoster in Taiwan.

Additional file 1 provides the flow chart of study cohort selection. For the first part of this study, we aimed to evaluate the incidence of herpes zoster among patients in all age groups. In the herpes zoster cohort, patients who had at least 1 inpatient or outpatient visit with a primary diagnosis of herpes zoster (The International Classification of Diseases, Ninth Revision, Clinical Modification (ICD-9-CM) code: 053.xx) during January 1, 2004 to December 31, 2008 were included and identified as herpes zoster cases. The index date was defined as the date of first diagnosis with herpes zoster between 2004 and 2008. Patients that had any diagnosis of herpes zoster within 1 year before the index date were excluded.

For the second part of this study, we aimed to examine the incremental disease burdens of herpes zoster among the elderly. We thus further identified those who were aged ≥65 years and with a diagnosis of herpes zoster. The rationale for selecting older herpes zoster patients was that this patient population was reported to be most vulnerable to herpes zoster and may incur more healthcare utilizations [8]. Another subset of the NHIRD, which contained randomly selected elderly beneficiaries (aged ≥65 years) between 2004 and 2008 and represented about 20% of the entire elderly insurers, was further used. The elderly cohort served as a control pool for identifying non-herpes zoster patients to match with our cases. In the elderly cohort, patients without a record of diagnosis of herpes zoster in outpatient or inpatient claims between 2003 and 2011 were included and their outpatient visits during January 1, 2004 to December 31, 2008 were further identified to serve as non-herpes-zoster controls. Individuals could be included multiple times if they had more than one outpatient visit. The index date was defined as the date of the outpatient visit between 2004 and 2008.

For all cases and controls, immunocompetent patients were included and those who had received a diagnosis of cancer (ICD-9-CM code: 140.xx–208.xx), HIV/AIDS (ICD-9-CM code: 042.xx), or had received any transplantation (ICD-9-CM code: V42.xx, 996.8x) in the one-year before the index date were excluded [15]. Furthermore, all study subjects were required to be continuously eligible to receive healthcare services for at least a 4-year observational period (1 year before and 3 years after entering our study cohort).

Among the herpes zoster cases, we further identified those who suffered from postherpetic neuralgia (PHN), defined as those with persisting pain for more than 90 days after the onset of herpes zoster [13]. Cases with PHN in our study were thus identified by having at least one diagnosis of PHN (ICD9-CM: 053.1x) during the 90 to 180 days after the index date [9].

### Propensity score matching

For each patient with herpes zoster, a matched control was assigned using the propensity score matching technique to account for baseline differences between herpes zoster and non-herpes-zoster patients [24]. The propensity score was assigned based on the probability that an individual would be a case of herpes zoster and estimated by a multivariable logistic regression model adjusting for observed covariates. Covariates included in the logistic regression model to generate the propensity score were autoimmune diseases (Additional file 2), chronic obstructive pulmonary disease (ICD-9-CM code: 491.xx, 492.xx, 496), chronic renal diseases (ICD-9-CM code: 250.4x, 274.1x, 283.11, 403.01, 403.11, 403.91, 404.02, 404.03, 404.12, 404.13, 404.92, 404.93, 440.1, 442.1, 447.3, 572.4, 580–588), depression (ICD-9-CM code: 296.2x, 296.3x, 300.4, 311), diabetes mellitus (ICD-9-CM code: 250.xx), Charlson’s comorbidity index (CCI), number of outpatient visits, number of emergency visits and number of inpatient admissions within 1 year before the index date [25, 26]. Each case was then assigned one matched control according to age (± 1 year), gender, index date (± 30 days) and propensity score (± 0.05 SD) [27].

### Outcomes

Several outcomes of herpes zoster population were measured in this study. First, annual incidence of herpes zoster from 2004 to 2008 were investigated and was defined as number of herpes zoster cases per 1000 person-year. In addition, the longitudinal and incremental changes of healthcare service utilization among individuals with herpes zoster and PHN compared to those without were estimated. The incremental changes were calculated as the differences between healthcare utilization within 1 year prior to and the first, second and third year (3-year follow-up period) post the index date among patients with herpes zoster, PHN and those without. The 1 year prior to the index date were defined as the baseline period. The healthcare service utilizations measured in this study including number of outpatient visits, number of emergency visits and number of inpatient admission.

### Statistical analysis

For descriptive epidemiological data, the trends in annual incidence were analyzed using Cochrane-Armitage trend tests [28]. Comparisons of categorical variables were conducted using the Chi-square test and McNemar’s test (matched data) while comparisons of continuous variables were conducted using the t-test and paired t-test (matched data). Furthermore, we have reported the absolute standardized difference between herpes zoster cases and controls for each variable to provide further information about the clinical meaningful differences [27]. Chow tests were used to examine differences in the time varying use of healthcare utilizations between herpes zoster cases and their controls [29]. All data management and analyses were performed using SAS 9.3 in Windows (SAS Institute, Cary, NC, USA) and a *p*-value < 0.05 was considered statistically significant.

## Results

**Fig. 1:**
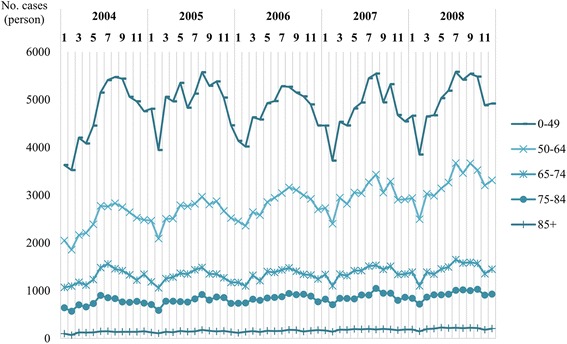
Seasonal variation in number of herpes zoster cases, stratified by age

Between 2004 and 2008, 601,090 patients were newly diagnosed with herpes zoster in Taiwan (Additional file 1). The estimated incidence of herpes zoster increased with age. For example, in 2008, the incidence was 3.59 in patients aged between 0 to 49 years and increased to 12.81 in patients aged between 65 to 74 years. The overall annual incidence rate of herpes zoster diseases raised from 5.04 in 2004 to 5.65 cases per 1000 person-year in 2008 (Table 1). Seasonal variation in number of herpes zoster cases was observed (Fig. 1). Most cases were identified during summers, but this seasonal effect became less pronounced among people in older age.

**Table 1:** Number of herpes zoster cases and annual incidence^a^

	2004		2005		2006		2007		2008		*p*-value for trend ^b^
	Cases	Incidence	Cases	Incidence	Cases	Incidence	Cases	Incidence	Cases	Incidence	
Overall	111,628	5.04	118,349	5.30	118,998	5.29	122,590	5.38	129,525	5.65	< 0.01
Aged 0–49	56,167	3.34	59,855	3.57	57,408	3.44	57,431	3.42	59,901	3.59	< 0.01
Aged 50–64	29,449	9.20	31,795	9.45	33,786	9.55	35,857	9.69	38,710	9.98	< 0.01
Aged 65–74	15,515	12.28	15,563	12.10	15,824	12.10	16,637	12.50	17,376	12.81	< 0.01
Aged 75–84	8938	12.58	9405	12.57	10,120	13.01	10,462	13.05	11,075	13.52	< 0.01
Aged 85+	1559	11.15	1731	11.39	1860	10.97	2203	11.81	2463	12.41	< 0.01

^a^Crude incidence rate: cases per 1000 person-year

^b^Trends in annual incidence were analyzed using Cochrane-Armitage trend tests [27]

**Fig. 2:**
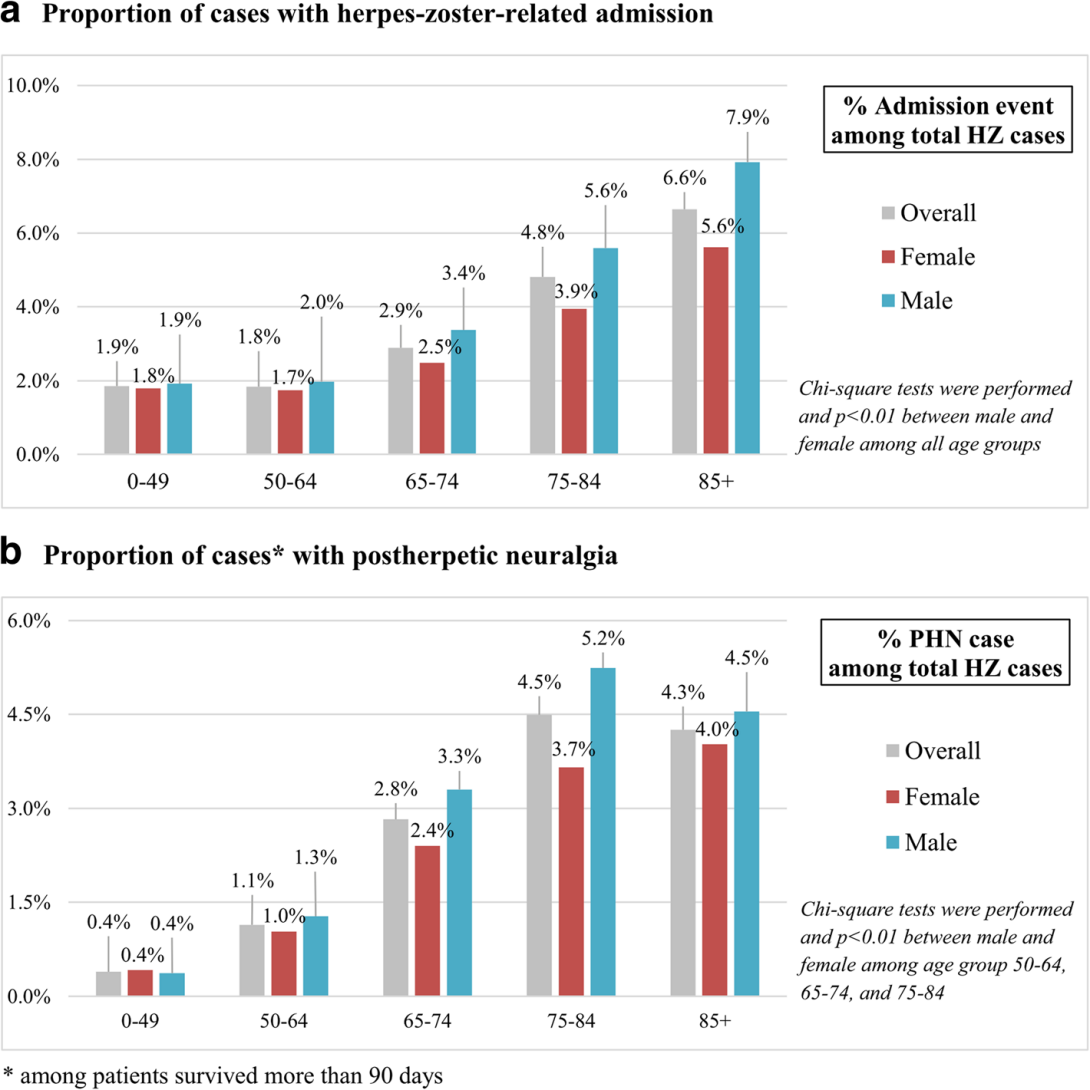
Gender difference of the disease burden in herpes-zoster patients. (**a**) Proportion of cases with herpes zoster-related admission. (**b**) Proportion of cases with postherpetic neuralgia

Compared to female patients, male patients were more likely to be admitted for herpes zoster care (Fig. 2a) and the majority of patients suffered from PHN (Fig. 2b), especially in the population aged 50 to 84.

Among patients newly diagnosed with herpes zoster, there were 140,731 patients aged ≥65 years at the diagnosis date (Additional file 1). After excluding patients with diagnosis of cancer, HIV/AIDS, or transplantation in 1-year period before the index date, patients without 3-year of follow-up, and patients without information of gender, the elderly herpes zoster cohort consisted of 115,105 patients. After applying the same exclusion criteria, 115,105 propensity score-matched controls were obtained based on their baseline characteristics and healthcare utilizations; comparisons between case and control groups after matching were shown in Table 2.

**Table 2:** Baseline characteristics of herpes zoster cases and matched controls

	Before matching						After matching					
	HZ cases (*N* = 115,202)		Controls (*N* = 34,506,574)		*p* ^a^	Absolute standardized difference	HZ cases (*N* = 115,105)		Controls (*N* = 115,105)		*p* ^b^	Absolute standardized difference
	*N*	*%*	*N*	*%*			*N*	*%*	*N*	*%*		
Age, years, mean (SD)	73.76 (6.15)		74.35 (6.01)		< 0.01	0.0973	73.75 (6.13)		73.65 (6.00)		< 0.01	0.0154
65–74	70,658	61.33	19,920,118	57.73	< 0.01	0.0734	70,639	61.37	71,849	62.42	< 0.01	0.0216
75–84	38,655	33.55	12,727,647	36.88		0.0698	38,622	33.55	37,685	32.74		0.0172
≥ 85	5889	5.11	1,858,809	5.39		0.0126	5844	5.08	5571	4.84		0.0111
Female	61,312	53.22	18,341,566	53.15	0.6	0.0014	61,262	53.22	61,262	53.22	–	0.0000
CCI, mean (SD)	1.21 (1.36)		1.51 (1.49)		< 0.01	0.2101	1.21 (1.36)		1.19 (1.33)		< 0.01	0.0132
0	44,044	38.23	10,025,484	29.05	< 0.01	0.1952	44,020	38.24	43,392	37.70	< 0.01	0.0111
1	33,894	29.42	10,363,659	30.03		0.0133	33,877	29.43	35,261	30.63		0.0262
2	19,481	16.91	6,757,478	19.58		0.0692	19,465	16.91	19,589	17.02		0.0029
≥ 3	17,783	15.44	7,359,953	21.33		0.1525	17,743	15.41	16,863	14.65		0.0213
Comorbidities												
Autoimmune diseases	14,647	12.71	5,256,307	15.23	< 0.01	0.0727	14,624	12.70	15,114	13.13	< 0.01	0.0128
Diabetes mellitus	25,247	21.92	9,368,723	27.15	< 0.01	0.1218	25,220	21.91	24,268	21.08	< 0.01	0.0202
CKD	8723	7.57	3,189,248	9.24	< 0.01	0.0602	8703	7.56	8400	7.30	< 0.05	0.0099
COPD	15,792	13.71	5,603,605	16.24	< 0.01	0.0709	15,765	13.70	15,048	13.07	< 0.01	0.0185
Depression	4516	3.92	2,017,321	5.85	< 0.01	0.0896	4502	3.91	4644	4.03	0.1	0.0061
Healthcare utilizations	mean	SD	mean	SD			mean	SD	mean	SD		
No. outpatient visits	25.66	21.26	36.56	26.98	< 0.01	0.4487	25.58	20.96	25.64	21.00	< 0.01	0.0030
No. ER visits	0.43	1.12	0.51	1.40	< 0.01	0.0621	0.42	1.10	0.42	1.25	0.6	0.0018
No. inpatient admissions	0.24	0.65	0.31	0.75	< 0.01	0.1011	0.24	0.65	0.24	0.63	< 0.01	0.0108

*CCI* charlson comorbidity index, *CKD* chronic kidney diseases, *COPD* chronic obstructive pulmonary disease, *HZ* herpes zoster, *ER* emergency room, *SD* standard deviation

^a^Chi-square test for categorical variables, t-test for continuous variables

^b^McNemar’s test for categorical variables, paired t-test for continuous variables

About 2.4% of herpes zoster cases acquired PHN after the primary herpes zoster disease (*n* = 2767). Compared to those without PHN, PHN patients were older (mean age 75.3 vs. 73.7 years) and had higher disease severity scores (mean CCI 1.38 vs. 1.21 of non-PHN). In addition, a higher proportion of PHN patients had concomitant diseases and higher consumption of healthcare services (Table 3).

**Table 3:** Baseline characteristics of herpes zoster cases with postherpetic neuralgia

	Herpes zoster cases						
	Overall (*N* = 115,105)		PHN (*N* = 2767)		Non-PHN (*N* = 112,338)		*p* ^a^
	*N*	*%*	*N*	*%*	*N*	*%*	
Age, years, mean (SD)	73.75 (6.13)		75.30 (6.11)		73.70 (6.12)		< 0.01
65–74	70,639	61.37	1358	49.08	69,281	61.67	< 0.01
75–84	38,622	33.55	1224	44.24	37,398	33.29	
≥ 85	5844	5.08	185	6.69	5659	5.04	
Female	61,262	53.22	1235	44.63	60,027	53.43	< 0.01
CCI, mean (SD)	1.21 (1.36)		1.38 (1.42)		1.21 (1.36)		< 0.01
0	44,020	38.24	901	32.56	43,119	38.38	< 0.01
1	33,877	29.43	828	29.92	33,049	29.42	
2	19,465	16.91	521	18.83	18,944	16.86	
≥ 3	17,743	15.41	517	18.68	17,226	15.33	
Concomitant diseases							
Autoimmune diseases	14,624	12.70	413	14.93	14,211	12.65	< 0.01
Diabetes mellitus	25,220	21.91	602	21.76	24,618	21.91	0.8
CKD	8703	7.56	236	8.53	8467	7.54	0.05
COPD	15,765	13.70	528	19.08	15,237	13.56	< 0.01
Depression	4502	3.91	130	4.70	4372	3.89	< 0.05
Healthcare utilizations, mean (SD)							
No. outpatient visits	25.58 (20.96)		30.37 (23.29)		25.46 (20.89)		< 0.01
No. ER visits	0.42 (1.10)		0.54 (1.15)		0.42 (1.10)		< 0.01
No. inpatient admissions	0.24 (0.65)		0.28 (0.67)		0.24 (0.65)		< 0.01

*CCI* charlson comorbidity index, *CKD* chronic kidney diseases, *COPD* chronic obstructive pulmonary disease, *ER* emergency room

^a^Chi-square test for categorical variables, t-test for continuous variables

**Fig. 3:**
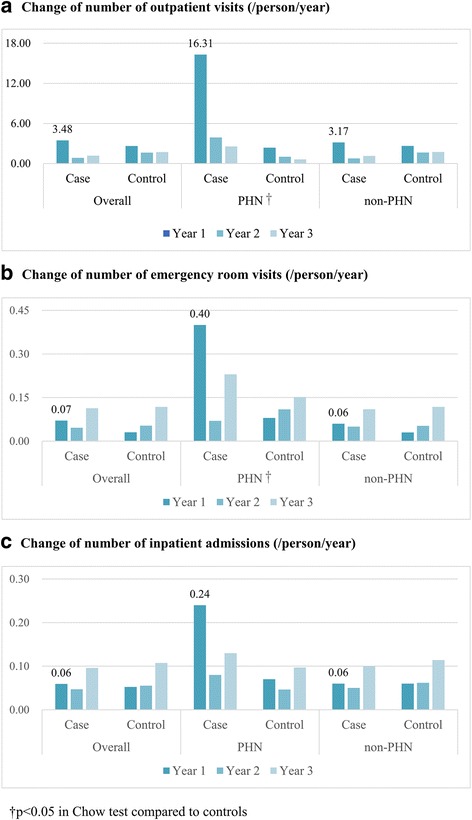
Incremental changes of healthcare utilization in the 3-year follow-up period. (**a**) Change of number of outpatient visits (/person/year). (**b**) Change of number of emergency room visits (/person/year). (**c**) Change of number of inpatient admissions (/person/year)

In the first year after index herpes zoster disease, herpes zoster cases were found to have had higher consumption in all types of healthcare services (including outpatient visits, emergency room visits and inpatient admissions). The significant increase in healthcare utilization during the first year was mostly attributed to patients with PHN, who increased about 16.3 outpatient visits, 0.4 emergency room (ER) visits and 0.24 inpatient admissions per year (Fig. 3).

## Discussion

This nationwide cohort study provided updated and important information about the epidemiology of herpes zoster and its most important complication, PHN. The incidence rate and economic burden of herpes zoster increased with age, especially among elderly patients. By application of the propensity score matching technique, we characterized the longitudinal and incremental change of the healthcare resource utilization among individuals diagnosed with herpes zoster and PHN compared to those without.

The overall incidence of herpes zoster in our study (5.04 to 5.65/1000 person-year during 2004–2008) is comparable to previous data in Taiwan (4.97 during 2000–2005) [9]. Compared to studies conducted at similar time points, the incidence rates in our study is higher than that in France (3.82) [10], the US (4.82) [30], and the Netherlands (4.75) [31], but lower than that in Germany (5.79) [32]. Our results also demonstrate that the incidence of herpes zoster has increased with age. These finding are consistent with previous studies in Taiwan [9] and other countries [30, 33, 34]. In addition, our finding that female accounts for a larger proportion of herpes zoster cases was consistent with previous studies [35–37]. However, by further investigating the healthcare utilizations and related outcome of these patients, we further found that male had more admissions for herpes zoster and suffered from PHN more than female especially in the population aged 50 to 84.

Seasonal changes in incidence rate of herpes zoster have been reported in a previous study. Wu et al. assumed the seasonal association between summer and the increased case number of herpes zoster was probably due to the change of temperature [38]. However, there has been no study that explored this phenomenon thoroughly stratified by age, and our study provides further information about seasonal change mainly observed in the younger population instead of the elderly.

After propensity score matching, herpes zoster cases were found to have higher consumption of all types of healthcare services within the first year after the index date. The increase was particularly obvious in patients with PHN. We further evaluated the incremental changes in outpatient visits for every 90-day period after the index date and found both the higher and longer needs of care in cases with PHN compared to their non-PHN counterparts (Additional file 3).

The proportion of herpes zoster patients who developed PHN in our study (2.4% in the current study) was lower than previous studies [39–41]. There are several potential explanations. Firstly, the estimates of the prevalence of PHN vary due to the different definitions of PHN. The definition we adopt was widely used in previous studies and usually result in conservative estimations of the case number of PHN. Compared to prospective studies which assessed PHN by direct questions from physicians, questionnaires, or visual analogue scale [34, 42, 43], our study using a specific ICD9-CM codes (053.1x) to identify PHN cases may underestimate the occurrence of PHN because of under-coding. In addition, there was no consensus on the duration of pain in PHN. Previous studies have defined PHN as pain persisting 30 days [39], 90 days [44], or 90 to 365 days [15, 45] following herpes zoster diagnosis. However, we only included specific PHN code recorded during 90 to 180 days post zoster to eliminate the chance of misclassification bias. The more stringent definition may result in the lower proportion of identified PHN among herpes zoster cases in our study. Secondly, some studies have demonstrated patients with immunosuppression status, including those using high doses of oral corticosteroids/other immunosuppressive drugs, having invasive cancer or HIV/AIDS, were significantly associated with higher risk of PHN [35]. However, in our study, those with diagnoses of cancer, HIV/AIDS, or receiving any transplantation in the one-year before the index date were excluded. The exclusion of higher risk patients may result in lower prevalence of PHN in our study. Lastly, prior study also suggested that herpes zoster patients with underlying conditions, such as diabetes, cardiovascular diseases, and respiratory diseases, have higher intensity of pain than those without underlying conditions [43]. Therefore, the comorbidity burden of the study population may also contribute to the difference of PHN prevalence between studies.

Although our study provides significant findings regarding the longitudinal burden of herpes zoster, this study has limitations, mainly due to the use of claims data. Firstly, we were not able to include variables not routinely captured in the claim database, such as smoking status, physical activities, frailty or daily pressure status. We have, however, attenuated observed differences by using the propensity score technique to create a match cohort. Second, patients with diagnoses of cancer, HIV/AIDS, or receiving any transplantation within one-year before the index date were excluded. We further required all patients to have at least a 3-year observational period after the index date. Therefore, our findings of longitudinal healthcare utilization may not be generalizable to patients with severe herpes zoster. Third, the ICD9-CM codes for PHN have been adopted based on the previous study [9]. However, the diagnosis codes may also include other severe nervous system complications and the estimation of healthcare utilization in our study may not be entirely comparable to other studies using different definitions of PHN.

## Conclusion

This nationwide cohort study provided updated and important information about the epidemiology and long-term disease burden of herpes zoster and its most important complication, postherpetic neuralgia (PHN). The incidence of herpes zoster increased with age and showed seasonal change. Herpes zoster patients were found to associate with higher healthcare utilizations compared to patients without herpes zoster and this increase was most obvious for herpes zoster patients with PHN.

## Additional files

Additional file 1: Flow chart of study cohort selection. (PDF 280 kb)

Additional file 2: ICD-9-CM codes of autoimmune diseases. (PDF 184 kb)

Additional file 3: Incremental changes of outpatient visit in days after herpes zoster infection (Year 1). (PDF 88 kb)

## Abbreviations

CCI: Charlson’s comorbidity index
CKD: Chronic kidney disease
COPD: Chronic obstructive pulmonary disease
ER: Emergency room
HIV/AIDS: Human immunodeficiency virus infection/acquired immune deficiency syndrome
HZ: Herpes zoster
ICD-9-CM: The international classification of diseases, ninth revision, clinical modification
NHIA: National Health Insurance Administration
NHIRD: National health insurance research database
NHRI: National Health Research Institutes
PHN: Postherpetic neuralgia
SD: Standard deviation
