# Supplementary material for: Epidemiology and long-term disease burden of herpes zoster and postherpetic neuralgia in Taiwan: a population-based, propensity score-matched cohort study
Source: BMC Public Health. 2018 Mar 20;18:369. doi: 10.1186/s12889-018-5247-6 (PMC5859733; doi:10.1186/s12889-018-5247-6)
Supplement: Supplementary file 1 — Flow chart of study cohort selection. (PDF 280 kb) [file 12889_2018_5247_MOESM1_ESM.pdf]

Additional File 1. Flow chart of study cohort selection

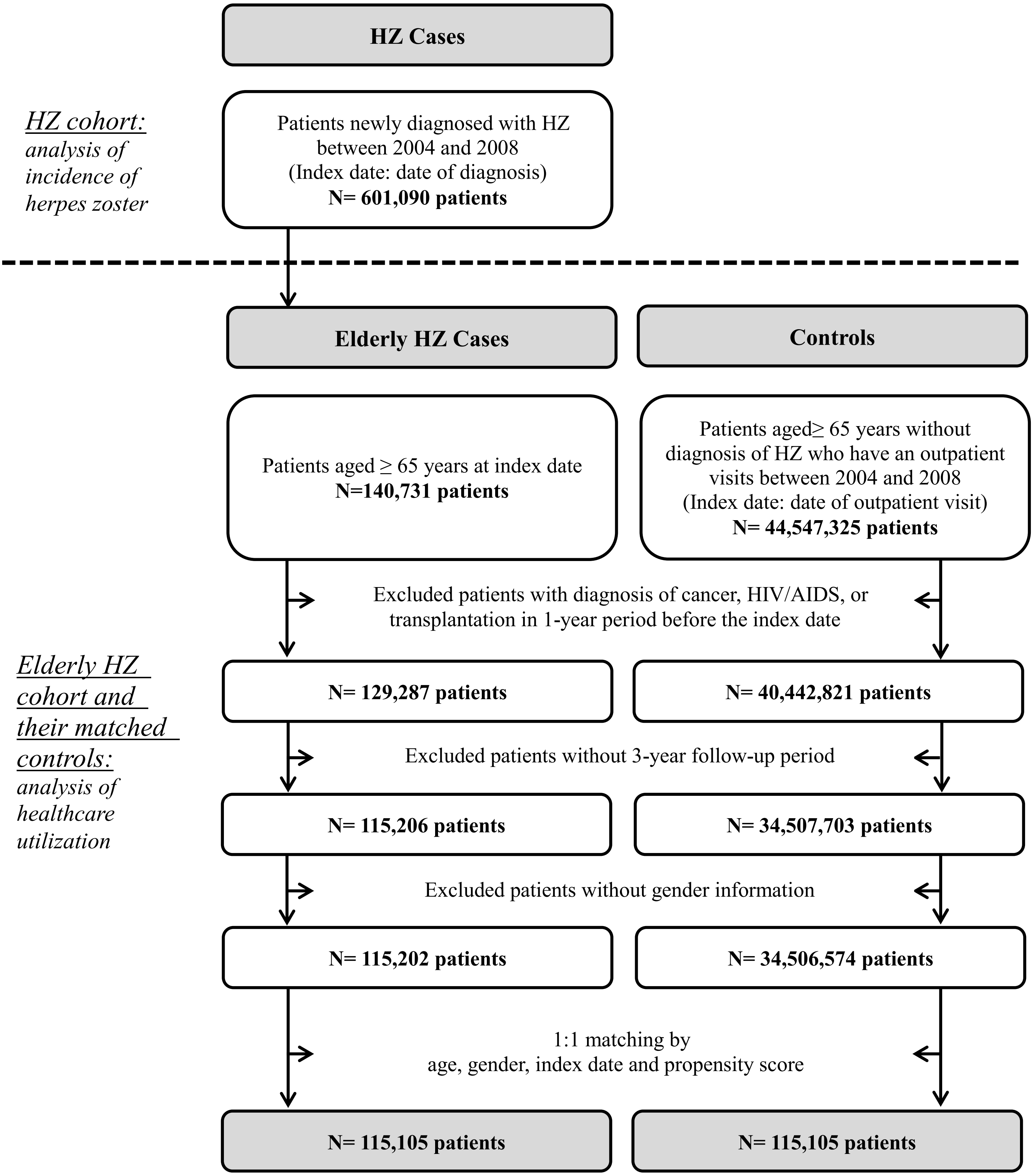

HZ= herpes zoster; HIV/AIDS= human immunodeficiency virus infection/acquired immunodeficiency syndrome.
