# Supplementary material for: Epidemiology and long-term disease burden of herpes zoster and postherpetic neuralgia in Taiwan: a population-based, propensity score-matched cohort study
Source: BMC Public Health. 2018 Mar 20;18:369. doi: 10.1186/s12889-018-5247-6 (PMC5859733; doi:10.1186/s12889-018-5247-6)
Supplement: Supplementary file 2 — ICD-9-CM codes of autoimmune diseases. (PDF 184 kb) [file 12889_2018_5247_MOESM2_ESM.pdf]

Additional File 2. ICD-9-CM codes of autoimmune diseases

Disease	ICD-9-CM code
Systemic lupus erythematosus	710.0
Scleroderma	710.1, 701.1, 711.1x, 778.1
Sjögren's syndrome	710.2
Polymyositis & Dermatomyositis	710.3, 710.4
Diffuse diseases of connective tissue	710.9, 279.4, 279.9
Rheumatoid arthritis	714.xx
Crohn’s disease	555.x
Ulcerative colitis	556.x
Vasculitis	136.1, 443.1, 446.0, 446.1, 446.2, 446.4, 446.5, 446.7, 447.6, 795.79
Pemphigus	694.4
Discoid lupus erythematous	695.4, 373.34
Psoriasis	696.0, 696.1, 696.8
Ankylosing spondylitis	720.0, 720.9, 716.9x
