# Supplementary material for: Epidemiology and long-term disease burden of herpes zoster and postherpetic neuralgia in Taiwan: a population-based, propensity score-matched cohort study
Source: BMC Public Health. 2018 Mar 20;18:369. doi: 10.1186/s12889-018-5247-6 (PMC5859733; doi:10.1186/s12889-018-5247-6)

Additional File 3.

Incremental changes of outpatient visit in days after herpes zoster infection (Year 1)

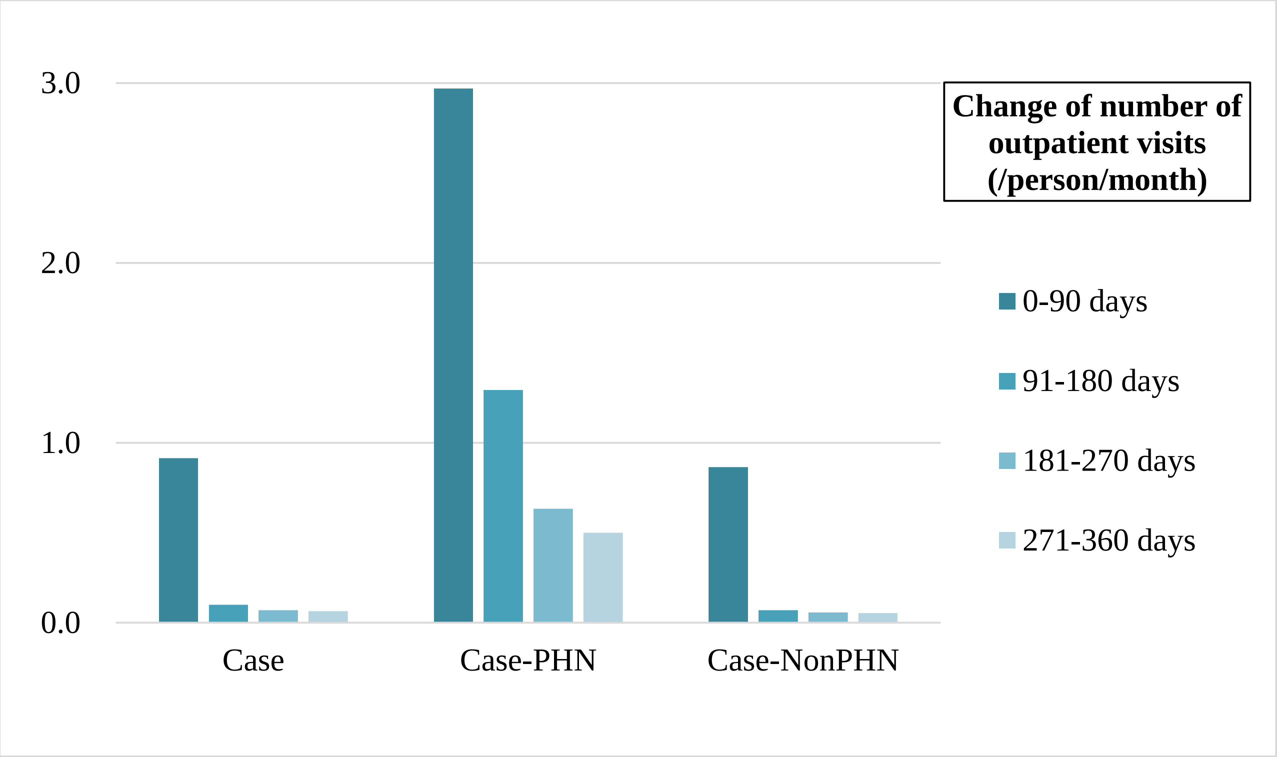

Supplement: Supplementary file 3 — Incremental changes of outpatient visit in days after herpes zoster infection (Year 1). (PDF 88 kb) [file 12889_2018_5247_MOESM3_ESM.pdf]
